# Higher and colder: The success and failure of boundaries in high altitude and Antarctic research stations

**DOI:** 10.1177/0306312716636249

**Published:** 2016-03-21

**Authors:** Vanessa Heggie

**Affiliations:** Social Studies of Medicine, University of Birmingham, Birmingham, UK

**Keywords:** altitude, Antarctica, experiment, exploration, field stations, fieldwork, laboratories, mountains, physiology

## Abstract

This article offers a series of case studies of field stations and field laboratories based at high altitudes in the Alps, Himalayas and Antarctica, which have been used by Western scientists (largely physiologists and physicists) from circa 1820 to present. It rejects the common frame for work on such spaces that polarizes a set of generalizations about practices undertaken in ‘the field’ versus ‘the laboratory’. Field sites are revealed as places that can be used to highlight common and crucial features of modern experimental science that are exposed by, but not uniquely the properties of, fieldwork. This includes heterogeneity of population and practice, diverse afterlives, the manner in which spaces of science construct individual and group expertise, and the extensive support and funding structures needed for modern scientific work.

## Introduction

This article challenges the utility of current understandings of scientific space that posit a binary division between the ‘laboratory’ and the ‘field’. This is not because place and space are unimportant or uninformative areas of investigation, but rather the reverse – the use of binaries is unnecessarily constricting, and we need a richer language of description to adequately understand the practice of modern science. The dangers of assuming a hierarchy of scientific practice and of linking experiment to the laboratory and practices like natural history to the field site have been highlighted elsewhere (Heggie, 2014; Strasser, 2009, 2010, 2011). Here, I present a series of case studies of particular field spaces and show how their historical users were not bounded by the assumptions of the philosophy or history of science. Furthermore, they demonstrate that studying what we currently consider to be field science can illustrate issues of relevance to what is currently designated as laboratory science.

In this article, I use the phrases ‘field laboratory’, ‘field station’ and ‘field site’ to indicate different spaces. A ‘field laboratory’ is a physical structure primarily intended for the *indoor* practice of science, while a ‘field station’ is a broader designation that may include domestic space, support structures and multiple individual laboratory spaces. ‘Field site’ is reserved to indicate the surroundings of the stations and laboratories, including the outdoor spaces in which scientific work is conducted. I do not intend these definitions to be absolute or prescriptive, but merely a way to identify with clarity the complex set of spaces discussed in this article.

The key role that the laboratory has played in the history of science has tended to result in its portrayal as the history of the embodiment – both rhetorical and physical – of the very principles of scientific practice and method themselves (Hannaway, 1986; Ophir and Shapin, 1991; Shapin, 1988, 1998). That is, laboratories are framed as co-constructors of the definition of science itself. For example, the laboratory is seen as an embodiment of the boundaries that scientific practitioners need or want to create between the uninitiated and the appropriate owners and creators of knowledge (Hannaway, 1986). In other cases, laboratories provide the isolation that scientists seek to claim a distance between their objective work and the social and cultural forces around them (Le Gars and Aubin, 2009; Shapin, 1998). Crucially, laboratories enable claims of control over environments, a particular feature of modern experimental practice (Gieryn, 2002). Of course, these instantiations are partial and debatable, but the historical role of the laboratory is nonetheless understood as part of an existing discourse of scientific practice within philosophy and sociology; laboratory science and ‘modern science’ seem inextricably intertwined.

It is this understanding that has informed the recent exploration and understanding of ‘the field’. Field sites and field stations are often framed as ‘others’, understood in opposition to the (platonic, if not real) ideal laboratory (Kuklick and Kohler, 1996). (The specific position of the field laboratory is perhaps more complex, although such spaces are rarely studied in isolation and are taken instead as part of a field station or field site.) The consequence of the assumption that laboratories are co-constitutive of what it means to do science or make modern scientific knowledge is that work in non-laboratory sites is often understood in two ways: either as a challenge to ‘laboratory-style’ practices or as an attempt to make field sites more ‘laboratory-like’ in order to make them trustworthy (Heggie, 2014). Alternatively, as Kohler (2002a, 2002b) argues, varieties of field sites may be considered ‘borderlands’ – although such a framing still sets the field and the laboratory in opposition to one another and invites understanding them through a series of binary notions: natural/man-made, artificial/real, domesticated/wild, controlled/unpredictable and bounded/unbounded (see also the reconsideration of the domestic by Opitz et al., 2015). Such structures are inadequate to describe modern science (Henke, 2000). But thus far the history of science has not responded to this critique (for notable exceptions, see De Bont, 2014; Vetter, 2012).

The relative neglect of field sites has shaped narratives told in the history of science. For example, the story of physiology in the 19th century is usually told as a triumph of the laboratory. Although the story is beginning to be revised (e.g. Finkelstein, 2013), the dominant narrative is that physiology was established as a legitimate member of the academy through the use of controlled internal spaces, reductive experimental models and an appeal to the new form of materialist anatomically focused medicine (Cunningham and Williams, 2002 [1992]; Schlick, 2007). The big names in this history are those who pioneered experiments (e.g. Francois Magendie, Claude Bernard) and/or precise measurement and mathematical rendering of biological phenomena (e.g. Justus von Liebig, Hermann von Helmholtz). One of the case studies considered in this article, pertaining to the Capanna Margherita, offers an alternative understanding. The work of Capanna Margherita’s main scientific founder, Angelo Mosso, demonstrates how oddly lopsided dominant accounts are when they fail to consider the work of actors in spaces other than the traditional university or private laboratory.

When field sites are considered, it is often as an expression of the intrinsically imperialist or colonial nature of scientific enterprise. This requires the control and domination of natural spaces in order to know them and to turn them to productive work creating knowledge, products, and trained (read: disciplined) scientists – such practices usually have explicitly national aims (Shen, 2009). This is generally framed as evidence of ‘laboratory’ discipline being applied to, or required of, the field. Yet, it is just as plausible to see these trends as originating from the field sites themselves; after all, the language of conquest, the gendering of mountains as female (and therefore natural and capricious), can hardly be claimed as a consequence only of the ‘rise of the laboratory’. It may well be that the presence or availability of such rhetoric is what makes some spaces appropriate sites for science and not others, but it is misleading to suggest that this rhetoric originates or has its obvious home in the laboratory. Likewise, field sites are often depicted as parts of hierarchical relationships, usually framed as centre-periphery; often, information flows are depicted as unilateral, with data collection in the field feeding into more and more centralized, abstract and metropolitan sites. In extreme cases, a single colonial scientist (e.g. Darwin, Newton) draws in a vast network of research from field sites and field workers (Schaffer, 2009). When field workers are given agency in this set up, it is generally limited to opposition to the centre/laboratory or to their participation in disciplinary processes (such as standardization or labelling), which the centre/laboratory is thought to impose on the periphery/field (Aubin, 2003; Bourget, 2002; Coen, 2009; De Chadarevian, 1996). Recent scholarship is beginning to challenge this unidirectional flow of power (Patiniotis, 2013). As this Special Issue – and this article – shows, field sites were often intimately connected with one another as well as, or even instead of, to ‘centres’; field sites’ own products could be so important that they became the centres of their own networks as they sent out samples, knowledge and trained scientists.

The high and cold field sites considered here are not isolated examples. Henke (2000) describes other situations where both the flow of information and the location of authority are sometimes inverted in periphery/field and centre/laboratory relations. Indeed, the field site often plays slightly different roles in works, particularly in sociology, that consider the use of the ‘field trial’ or similar forms of semi-public test and display (Latour, 1988). For example, Millo and Lezaun’s (2006) examination of the role of ‘regulatory experiments’ in the construction of political policies uses field trials to bridge the ‘experimental gap’ between ‘the world at large’ and laboratory or theoretical models (p. 181). I have already discussed the ways in which several of the sites here bridge the ‘experimental gap’ (Heggie, 2013), and I will only touch upon them in this article, but it is worth noting that the field sites discussed here all involve various publics (particularly mountaineers, explorers and funding bodies) and performances (of ‘successful’ expeditions, nationality and masculinity). We should not be lulled into thinking that this bridging work is a special characteristic of the field site. It is possible to treat every laboratory as a field site; however, attempts to reconsider the relationship between fields and laboratories have thus far still tended to maintain an essential divide between the two sites (Vetter, 2012).

All the spaces of science are unique; it is in the practices of science that we find both the unique and generalizable properties of scientific spaces. It is clear that the existing stories about these spaces are being reconsidered by many historians, but it is not clear whether there will emerge from this reconsideration a categorization that meaningfully shows how the set of field sites is significantly more alike, or shares more common features, than the set of non-field sites. Work done so far seems to suggest the contrary: take, for example, David Aubin’s (2009) analysis of the ways in which mountains (specifically Alpine mountains) could be justified as valid and important homes for science in the 19th century. First, they may be sites with special features that deserve specific scientific attention; second, they may be considered or constructed as conveniently knowable microcosms representing harder-to-study larger environments; finally, they may be ‘macro-tool[s] for the pursuit of science’ (p. 365). All three of these explanations also apply to the case studies discussed here, but they are such broad categories that they surely apply to any site of scientific practice. University administrators have been heard to claim that a particular university laboratory offers unique opportunities for research due to transport links, local industry and also a diverse and willing population available for medical or social science research. Such university laboratories are also absolutely intended to act as microcosms of natural phenomena, and when used in certain ways they can be macro-tools, spaces which provide particular environmental conditions conducive to certain investigations. It is not clear whether these three functions, derived from a field site case study, cannot be ascribed to *any* laboratory, *any* ‘home of science’ *anywhere* in the world at *any* time in the modern period.

The case studies presented in this article – of Alpine, Himalayan and Antarctic laboratories – are a gesture towards the richer, and therefore more precise, landscape of the homes for science we can create. First, they demonstrate that there are times when field site workers seem to find no challenge at all in seamlessly transgressing boundaries that historians wish to ascribe to scientific practice: sometimes indigenous and foreign, public and private, wild and domesticated, museum science and laboratory science present more problems for the historian than for the historical actor. Second, these case studies show how field sites are useful spaces for historians who want to find out about the practice of modern science. Far from being unusual research spaces – ‘others’, counters to the trend to the laboratory or exceptions to a process of modernization – field sites embody trends that have been previously ascribed as the special properties of the traditional laboratory. Fields can help us understand issues of isolation, funding, community formation and the mixed uses to which scientific spaces can be put. If there is a unique feature that meaningfully distinguishes field from non-field sites, it may only be that scientists working in the latter are more likely to explicitly articulate ideological and philosophical positions about science, particularly about the distance between nature and artifice (Heggie, 2013).

This is not to make the case that space does not matter in the light of practice, but rather to show, first, that ‘placeless places’ are not essential to truth-making, and second, to point out that when placelessness matters, it has to be constructed by traditional laboratories as much as by field sites, as Gieryn (2008) has clearly demonstrated. Third, then, this article argues that the heterogeneity of field sites (and by implication, non-field sites) needs to be better acknowledged. Individual field laboratories may be extremely unalike as well as closely related; some may depend on a hierarchical relationship with non-field sites, while others may be an authoritative and dominant source of truths about the natural world. Connections between field sites can be physical and material, involving the movement of ideas, people, objects and funding. They can also be symbolic and representational, such as the deliberate recreation of an expedition, claiming a famous field laboratory as an ‘ancestor’ of a modern study, using field sites as obligatory passage points, or even conceptualizing field laboratories as sites of secular pilgrimage. What these heterogeneous sites have in common is that it is not possible to understand their roles in isolation. All were part of networks as individual scientists moved between multiple field and non-field sites and because of that the sites themselves are reinvented – even physically relocated – to achieve different scientific goals. A unitary (making placelessness), binary (lab/field) or even ternary (lab/borderland/field) system is not sufficient to analyse these processes.

## Never pure? Physiological research and the alpine laboratory

Mountains have been the sites of natural, philosophical and scientific fact-making for centuries; Florin Périer’s mid-17th century trip up a long-dead French volcano to test Toricelli’s ideas about air pressure for Blaise Pascal is perhaps the earliest famous example. While modern researchers have explicitly referred to mountains as ‘natural laboratories’ in and of themselves (Hackett, 1988; West, 1985), it is only from around the mid-19th century that buildings on mountains began to be constructed or appropriated for the specific purpose of scientific research. Among the first of these, according to David Aubin (2009) was a chalet built in 1823 on Mount Faulhorn in the Swiss Alps. This was ‘one of the first semi-permanent mountain observatories in the world, by which [Aubin means] this was a fixed site on the planet where observations of any kind were repeatedly made over a period of time’ (Aubin, 2009: 367). This chalet, whose primary purpose was to offer hospitality to climbers, walkers and tourists, was later enlarged into a hotel. Among these visitors were many engaged in work relating to meteorology, earth and air sciences, geography and geology, and even nutritional science, as well as astronomy.

There is a strong trope in histories of European laboratories in this period – particularly astronomical observatories – that points to the increasing isolation of research sites, as scientists fled critical publics as well as the noise and light pollution of industrializing cities (Coen, 2009; Le Gars and Aubin, 2009). Although retreating to a mountaintop might seem to fit this pattern, on closer investigation it is clear that the Faulhorn does not neatly echo the story of isolation: the mountain was popular not because it was isolated but because it was accessible, and it had a hotel at the summit serving hot drinks and offering warm beds. Given the vastness of the Alpine region where scientific work could have been done in perfect isolation, the fact that a peculiar concentration of scientific men^1^ passed over the Faulhorn suggests that its connectedness had some particular appeal. Similar choices were made by scientists in the 20th century: for example, JS Haldane explicitly chose Pike’s Peak in the United States for his own high-altitude research because it had a cog-train to the summit (West, 1998: 114). One might better understand these mountains and their huts and hotels as early modern coffee houses or scientific society meeting rooms; they were places with restricted access that afforded comforts and like-minded companionship to participants, rather than isolated refuges for individual intellectual endeavours.

**Figure 1.**
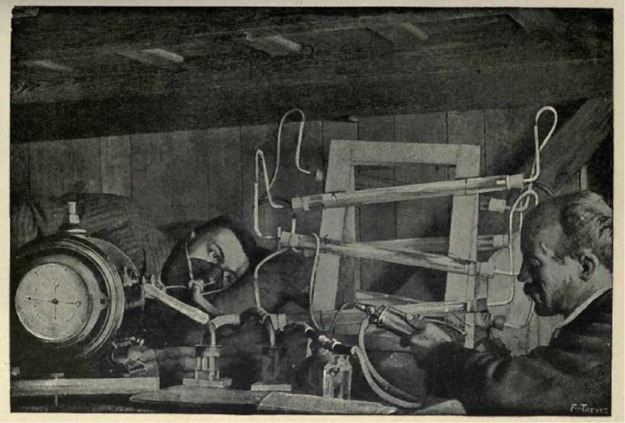
Taken from Mosso (1898).

Elsewhere in the Alps, other hotels, as well as huts, bothies, caves and crags, became places for both organized and impromptu scientific work. Some sites were appropriated, like the Faulhorn hotel, from other functions, while others were deliberately built or redesigned to be homes for science. The highest site specifically and primarily designed for scientific work appeared at the very end of the century, in 1893, with the opening of the *Capanna (Regina) Margherita* (the Margherita Hut) near the summit of Monte Rosa in the Italian Alps, around 4450 m above sea level (West, 1998: 81–82). The funding for the hut came in part from Queen Margherita of Italy (after whom the hut was named) who was persuaded to sponsor the build by Italian physiologist Angelo Mosso. The Queen – a keen climber herself – opened the hut in August 1893. At that time it consisted of a kitchen and living space; a laboratory was added in 1894, and the whole hut extended in 1898 to create more space for research. (The cramped facilities can be seen in Figure 1.)

Although the hut was used sporadically for meteorological and astronomical research, Mosso’s involvement meant that the Capanna Margherita was a key site for physiological research. There is no good, comprehensive outline of his research activities in English (more on why this is the case below), but Philip Felsch (2007) provides a fairly exhaustive outline in German. Mosso’s work in and around the hut generally divides into two strands. First, he researched fatigue, wanting to find out whether it was a physiological, chemical or psychological process. For this purpose, he used the hut as a resting point for his human guinea pigs (usually mountaineers or soldiers) and a site for analysing their bodies. Second, he established a wide-ranging experimental programme into all aspects of life and survival on mountains and at altitude, which included serious investigations into mountain sickness, the most effective diets for physically active bodies, emergency medical treatments and even attempts to design effective sunblocks.

**Figure 2.**
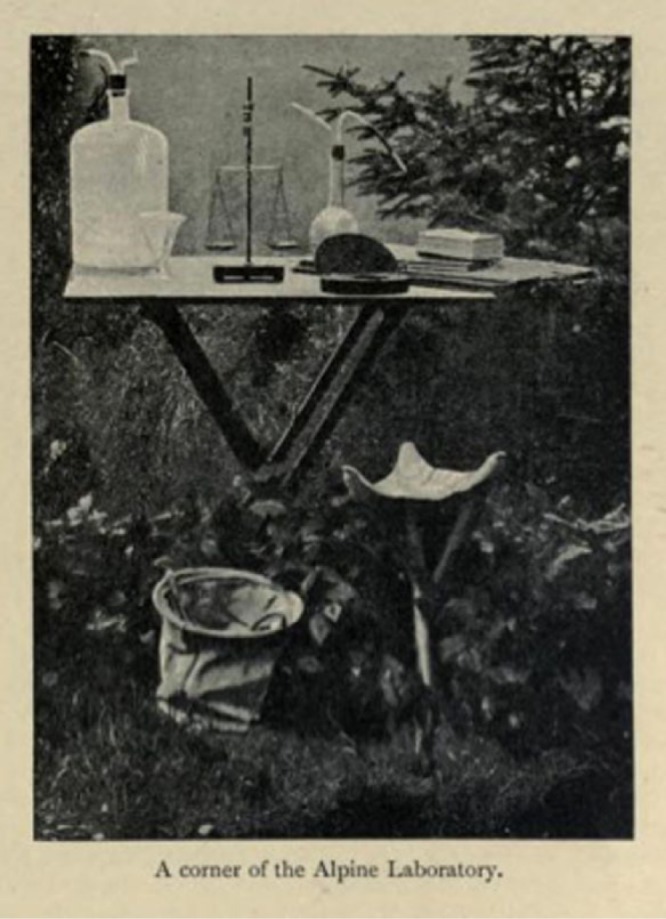
Taken from Mosso (1898).

Mosso’s major publication on altitude studies, *Fisiologia dell’uomo sulle Alpi* (1897),^2^ contains an outline of much of this research. It also makes clear that the Capanna Margherita was not an isolated outpost for research but instead a key node in an eclectic set of work sites that Mosso used. These included his traditional sea-level university laboratory, various mountainsides with and without their own field stations and expensive technological ‘macro-tools’ such as the barometric chamber; this network was interlaced with the researches of other scholars across Europe, as he cites work done by other scientists on Monte Rosa, as well as their laboratory and abstract mathematical studies. Mosso explicitly refers to the mountainside as a ‘laboratory’ (see Figure 2), but a mountain might also be an abstract concept, best modelled using expensive machines in sea-level laboratories. Equally, this technology might be a second-best alternative to the mountain, such as in his experiment on the effect of low air pressure on a teenager with a hole in his skull (Mosso wanted to take his subject up the ‘real’ mountain, but the boy refused to go). Both sea-level laboratory and mid-altitude field station could be turned into domestic and even agricultural spaces, for the care (and breeding) of experimental animals, whether mountaineers or marmots.

It is difficult to express the range of Mosso’s activities: from carefully placing hibernating marmots under pneumatic bells, manipulating human corpses to calculate the effect of wind on air pressure in the lungs, to taking meticulous traces of his own pulse, Mosso’s work is a collage of methods, approaches and spaces. Not only were all the sites he used interdisciplinary ones, but they were all clearly populated with a diverse range of actors: suspicious local apprentices who had suffered axe-related head injuries, animals of all kinds, professors from across Europe, soldiers, agriculturalists and students all participated in the making of Mosso’s scientific facts. This diversity is represented well by the Hut itself, which was a collaborative enterprise between Mosso, the Italian Alpine Club, the Italian Royal Family, the Italian army, and local artisans and mountain guides. There is little, in Mosso’s printed works at least, which gives any sense that this heterogeneity, these porous boundaries between sites, these blurred dichotomies (lab/field, experiment/experience), gave him any intellectual or philosophical trouble at all (Felsch, 2007).

The Capanna Margherita is an excellent example of the richness I highlight in the field sites of the long 20th century; it plays a role both similar to and distinct from any traditional laboratory space. The hut was there for practical physical shelter, providing a place to sleep, and to make and store food and climbing equipment. It was a place for the replication of experiments using equipment and techniques identical to those at sea-level traditional laboratories, to either confirm or deny the importance of place in biological phenomena. At other times, it was a special site that provided unique access to knowledge about the natural world, or was the *preferred* site for knowledge production, which could nonetheless be adequately modelled using technology (such as the barometric chamber). It was isolated and connected, unique and replicable, domestic and professional. And yet, Mosso’s full portfolio of work rarely features, particularly in English-language publications, in the history of science. He is mentioned only in relation to his work on fatigue and in particular his invention of the ergograph, a machine intended to convert the subjective feeling of fatigue to a graphical representation that could be measured and mathematized (e.g. Rabinbach, 1992; Tuccimei, 1987). The obvious reason for this spot-blindness is the dominance, as discussed above, of the rise of the laboratory as a narrative for the history of (life) science in the 19th century (Cunningham and Williams, 1992 [2002]; Schlick, 2007). In that narrative, Mosso’s ergograph has an obvious place, but not so his other research: not only was his work interdisciplinary, blurring boundaries between physiology, medicine, physics, anatomy, and even meteorology and anthropology, but his workspaces were equally eclectic and his working practices not convenient evidence of a rise of reductive empiricism.

The Margherita Hut is a microcosm of a more diverse world of late-19th and early 20th-century scientific work, which is just beginning to be revealed by a new generation of historians of science (De Bont, 2014; Finkelstein, 2013). This is not to suggest that the Hut did not have its critics or its own limitations. It was still an Alpine hut, albeit a very expensive and carefully designed one. In 1920, researchers at the Physiological Laboratory of Cambridge University wrote about the limitations of existing field stations, pointing out that ‘[e]ven the Margherita Hut’ (which was clearly considered by them to be an advanced example of the type) ‘is but an improvization as compared with a modern physiological laboratory’ (Barcroft et al., 1920: 453). This is a common representation of field laboratories, which often appear in a subordinate position or, when compared to ‘normal’ laboratories, as lacking equipment or domestic comforts. And yet, of course, field laboratories must also always possess features that are lacking in non-field laboratories, otherwise there could never be a justification for their use. Such justifications are amply recorded in scientists’ writings, especially in grant applications and pleas for funding, but are more rarely acknowledged by historians. We can read between the lines: the criticism of the Margherita Hut in 1920 was part of a contentious and long-running debate about lung function (Milledge, 1985), and, as other studies have shown, when experimental results differ, the location and skill of the opposing experimenter are often a target for criticism (Collins, 1985).

Whatever its limitations, the Capanna Margherita continued to be a site of active scientific research through the 20th century, although by the mid-20th century such work was more commonly related to physics – such as the study of cosmic rays (e.g. Hintermann, 1954) – and meteorology than to physiology. It was renovated in the late 1970s, which led to a reinvigoration of its role as a base for physiological experiments. Consequently, the Capanna Margherita (with the newly named Mosso Laboratory) accrued a new identity in the 21st century as a ‘staging site’ for scientific research at much higher altitudes. The Caudwell Xtreme Everest Expeditions and subsequent medical expeditionary teams from the Centre of Altitude, Space and Extreme Environment (CASE) Medicine from University College London used the renovated hut as a second test site for suitability for experiments prior to full expeditions to Everest. Proposed studies were first checked in sea-level laboratories in the United Kingdom, and if found suitable taken to the Capanna Margherita. There they were re-assessed for suitability, and the most successful taken on the Everest expedition (Heggie, 2013). Here, the field station performs a new role for science: first, it is an important and limited resource and needs to be conserved by ‘pre-testing’, but in turn it provides a similar gate-keeping function for the even more exclusive experimental site of Everest.

## Never isolated? From Antarctica to the Silver Hut

Capanna Margherita illustrates three features shared by all of the field sites considered in this article. It is highly networked (to laboratories across Europe, as well as connecting London sites of science with Everest), it hosts an international group of researchers and it is regarded with nostalgia by contemporary scientists. This last point will be addressed more below, when we consider the afterlife of one of the most iconic field sites in physiology, the sites associated with the ‘Silver Hut’ expedition of the early 1960s. Silver Hut was an experiment in high-altitude physiology, but it drew as much from Antarctic research as from previous high-altitude studies, so it too epitomizes the interdisciplinary, international and networked nature of the work of modern science, shown here to be as visible in field sites as in traditional laboratories or networks of correspondence across Renaissance Europe.

The silver-coloured hut itself was designed by the architect Ezra Levin with help from the British physiologist Lewis Griffiths Creswell Evans Pugh (‘Griff’ Pugh).^3^ When assembled in 1960, it became the highest laboratory structure in the world, located more than a kilometre higher than the Capanna Margherita, at 5800 m above sea level on the Mingbo glacier, about 20 km south of Mount Everest. Among mountaineers and altitude researchers it is highly celebrated, a landmark in altitude medicine and physiology whose research is usually claimed to have remained unrefuted after more than half a century of subsequent study (Milledge, 2010). Outside that circle it is almost unknown, especially to historians; while the practitioner accounts tend to the celebratory and the mountaineering accounts tend to the heroic, a pioneering biography by Harriet Tuckey of her father, Griff Pugh, has begun to reveal the fractious, difficult and sometimes dangerous history of this expedition (Tuckey, 2013).

The two leads on this research project were the physiologist Pugh and the mountaineer Sir Edmund Hillary. Both men had been involved in the successful attempt to climb to the summit of Everest in 1953, an achievement in large part due to Pugh’s field-based research works the previous year on Cho Oyu (about 20 km west of Everest), where he worked on the technical and behavioural changes needed to get climbers and their oxygen kits to the highest point on the earth’s surface (Tuckey, 2013). Subsequently, both men also went to Antarctica. Hillary went with the Commonwealth Trans-Antarctic Expedition of 1955–1958, a British Commonwealth-funded expedition led by Sir Vivian Fuchs that aimed to complete the first overland Antarctic Crossing (Dodds, 2005). Pugh went as part of the International Physiological Expedition to Antarctica, or INPHEXAN, to use its US military designation.^4^ The six-man INPHEXAN team represented three nations: Pugh was joined by British Army medical officer Major James Adam; from the United States came the Commanding Officer of the US Naval Medical Research Unit No.1, Lt Cmdr. Jack W Millar, expeditionary biophysicist William Siri from Berkeley and the expedition’s organizer, physiologist (and Naval Reserve officer) Nello Pace; the sixth team member, on special invitation by Pace, was German physiologist Gerhard J Hildebrand.

INPHEXAN did not build any independent field stations; they made use of the existing, often military, infrastructure around Hut Point, Scott Base and at other locations in the Antarctic, and collaborated in the building of new homes for science; this field site extended fully to the South Pole. With the assistance of Fuch’s team physiologist (Dr Alan Rogers), blood and urine samples and temperature measurements were made on the crossing teams, to compare to control samples taken from the British members of the team back in the United Kingdom. It was Siri who flew in to the South Pole to collect these samples, and he and Hildebrand also collected further biological samples by joining the Ross Shelf traverse party for three days in late December.^5^ Work was also done at the Amundsen-Scott South Pole Station and on the Victoria Plateau, where field stations were built in collaboration with researchers already in the Antarctic for the International Geophysical Year.^6^ This interdisciplinary work included geophysical and meteorological studies, taking ice cores, conducting a bacteriological survey, and zoological work with seal blood samples. Pugh’s own research focused on physiology, notably cold survival, which included changes in the bodies of explorers as well as studies of clothing, accommodation, the effects of wind and radiation and so on. Although many of the samples were returned to Berkeley for testing, some went back to the Medical Research Council’s Division of Human Physiology in the United Kingdom, where Pugh did further work on them. (This involved studies of the effects of solar radiation on clothing insulation in Switzerland, connecting the Antarctic to the Alps).^7^

In 1957, Pugh suggested to Hillary that they should return to the Everest region to continue studying altitude physiology and human adaptation to altitude. Furthermore, he suggested, they should copy the established mode of polar fieldwork that both men had experienced in Antarctica. This is what Pugh referred to as ‘overwintering’ or the building of a semi-permanent infrastructure that allowed Antarctic visitors to stay through the coldest parts of the year in order to extend the possible period of research. It was also an experimental methodology: by facilitating a long stay at altitude, the expedition could study long-term adaptation to high altitude in sea-level residents, a research puzzle that had long led to the prioritization of the field site over laboratory or bench-top models (Heggie, 2013). Previous studies of long-term adaptation had instead tended to examine permanent, long-term, or indigenous residents at high altitude in South America (Tracy, 2012). Pugh was also considering the possibility of a ‘purely scientific’ expedition of eight members to Mount Kamet, a remote Himalayan peak on the border between India and Tibet, from March to June 1959.^8^ This was abandoned after Hillary agreed to Pugh’s proposal for an expedition to Everest; Pugh hoped that the ‘overwintering’ scheme would allow for the first attempt on the world’s highest mountain without bottled oxygen (Tuckey, 2013: 190).

In the end, the Silver Hut Expedition (or, as it is formally known, the 1960–1961 Himalayan Scientific and Mountaineering Expedition) was designed with three aims, including Pugh’s two purposes of researching into the long-term adaptation of lowlanders living at high altitude and supporting through this research a possible attempt on Everest. To these aims Hillary added a third, a hunt for the Yeti, in order to raise funds from the Field Enterprises Educational Corporation, a text-book publisher in Chicago. The ascent of Everest never happened, as, for complicated reasons that are explored elsewhere (Tuckey, 2013), permission was not granted for climbing in the Everest region, so instead a plan was drawn up for an attempt on Makalu, the world’s fifth-highest mountain. This attempt failed and had tragic outcomes. I probably do not need to add that the Yeti was not discovered. But Pugh’s research programme became the celebrated, successful and extremely productive ‘Silver Hut’ project.

As a home for science, Silver Hut represents several quite different spaces. This nickname apparently derives from the name given to the shiny main laboratory building by some of the Sherpa people on the team. That this is the nickname indicates that the whole expedition – even in formal publications in scientific journals – is a mark of the importance of physical structures to expeditionary science.^9^ In scientific articles, newspaper pieces, historical work and the informal letters written by the team members, ‘Silver Hut’ is not a consistent site: it is the nominal silver-coloured research hut; it is also the broader field station including the accommodation hut (Green Hut) built at lower altitude; it is the entire field site including the open spaces of the Mingbo Glacier and the surrounding Himalayan peaks where mountaineering and research fieldwork took place; it is specifically the physiological research programme; or it is the entire scientific programme, including meteorological, geological and (in the case of the Yeti hunt) zoological work; it is the whole expedition, including the successful and disastrous mountaineering trips. This slippage makes clear the interdependence of all parts of this expedition: each piece of the research, whether a stint on a stationary bicycle in a specially designed laboratory hut, a pulse reading taken on a mountain slope, or a personal report of the experience of mountain sickness on a crag nearby, was imbricated with all the other activities of the trip. The laboratory could not function without the high-altitude location, without the Sherpas to carry it up the mountainside, without the climbers to co-fund and co-run the experiments, without Green Hut to host the scientists and their research subjects, or without the Yeti to bring in funding.

Although not as complicated as the intersecting sets of work and experiment done in the Antarctic, the Silver Hut expedition was also a web of activity involving shifting groups of personnel. While Hillary led his Yeti hunt, British physiologist James (‘Jim’) Milledge, Americans Barry Bishop (geographer) and Willy Romanes (mountaineer with building experience) and New Zealand mountaineer and civil engineer Norman Hardie went with 310 porters to the Mingbo Glacier and erected the Green and Silver Huts. Milledge, Bishop and Romanes remained to be part of the overwintering scientific and climbing team, joined by Pugh, Australian physiologist John West, New Zealand climber and medical student Michael B Gill, US Air Force doctor Tom Nevison, Oxford-trained Indian physiologist Sukhamay Lakhiri and Indian Army Medical Corps Captain S. B. Motwani. Gill, Romanes, West and Milledge went on to join Hillary’s attempt on Makalu, a disastrous attempt during which Hillary suffered a stroke and which resulted in the eventual amputation of both the feet of New Zealand climber Peter Mulgrew. Team members therefore had multiple identities, as some were part of climbing parties and research teams, and many acted as guinea pigs; this included some of the Sherpas, who were also guides, technical advisors and domestic assistants.

**Figure 3.**
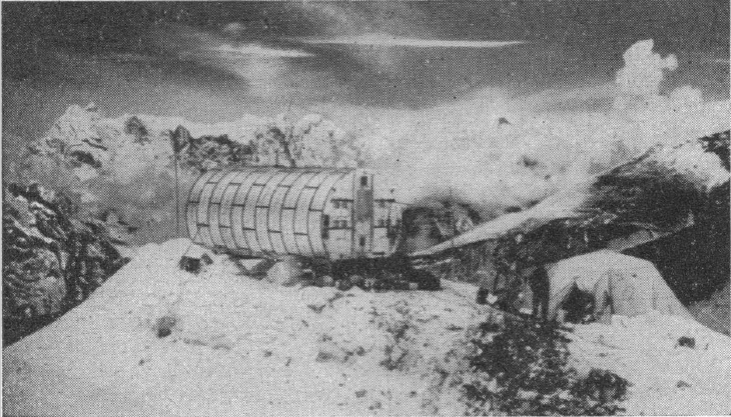
The ‘Silver Hut’ in its original position on the Mingbo Glacier (Pugh, 1962: 622).

The laboratory hut itself (see Figure 3) was made of curved segments, so it could be disassembled into individual ‘loads’ for porters; at 6.7 m long, 3 m wide and a little under 3 m high, it looked a little like a ‘London underground train carriage’ (Milledge, 2010: 94). Inside, in addition to a stove and bunk beds, was an exercise bicycle and the trappings one might expect in a university physiology laboratory, including delicate equipment such as the Lloyd-Haldane apparatus used for analysing gases (two of these had been smashed on the trek in and replacements had to be hurriedly requested). The physiological work done in the laboratory and across the Silver Hut field site was diverse, although all focused largely on the question of adaptation to, or sickness caused by, altitude. This includes studies of exercise at altitude, considering changes in VO_2_max, blood oxygen levels, general fitness levels and so on; body composition and metabolism were routinely measured and the rate of excretion of metabolic products in the urine examined; subjective accounts were made of the experience of living and climbing at altitude; cold resistance studies were made; and card sorting and other tests were deployed to examine the effect of altitude on cognition and information processing.

I do not mean to present a picture of seamless boundary crossing and interdisciplinary and multi-site work; it was not unproblematic for spaces to be both domestic and professional (although Green Hut was the main site for domestic processes, eating and sleeping happened in Silver Hut too). For example, one experiment was disrupted because of the misuse of a primus stove in Silver Hut, which caused several of the team to suffer from carbon monoxide poisoning.^10^ Not all of the experiments were successful, and not all of the guinea pigs passively amenable: there was particular issue with persuading the Sherpa subjects to take part in bicycle ergometer tests (Tuckey, 2013: 212). There was also an extraordinary clash between Hillary’s climbing ambitions and Pugh’s scientific programme, only resolved when Pugh wrote to several funders threatening to withdraw from the expedition, as he feared Hillary’s proposals were an ‘act of “gross negligence”’ (Tuckey, 2013: 216). It is important to note that this was a clash of real personalities, not one between the abstractions ‘sport’ and ‘science’; there has been a tendency in sports history to represent (British and Commonwealth) sporting ethics as at best ambivalent about, and at worst actively opposed to, scientific and biomedical intervention. This is particularly the case for mountaineering, but it is sometimes a misrepresentation – indeed, another unhelpful binary set up by historians and not respected by historical actors (Heggie, 2013).

These fractures and accidents are largely in visible in the scientific literature relating to Silver Hut; data were produced, analysed and written up in seamless documents with multiple authors that contain no traces of controversy. While this may be partly put down to the effects of scientific publishing effacing the ‘work’ and messiness of scientific practice, it is worth pointing out that all the participants in the scientific projects maintained relationships with one another, collaborating with and celebrating each other on multiple occasions. (The full list of publications is too great to be recorded here, but for examples, see Gill et al., 1962; Gill and Pugh, 1964; Milledge, 1963; West, 2010). The problems are instead visible in the archives, and they can be read between the lines in the mountaineering autobiographies written by Hillary (2003), Mulgrew (1981) and others. The overwintering team was remarkably *un*fractious (the major issue seems to have been Hillary, not the scientific work), especially given the diverse research interests, nationalities and origins of the nine men who worked inside the Silver Hut laboratory.

Because of the isolation and potential ‘cabin fever’ of field laboratories, thought is often given to the make-up of the research and expeditionary parties. Researchers have taken friends, colleagues and family members whom they trust (crucial in environments that pose genuine threats to life) and whose company they feel they can tolerate for long periods of time. The importance of ensuring a balanced team, promoting harmony by only taking ‘good chaps’, is easily visible in discussions of polar expeditions and mountaineering trips (Gilchrist, 2008) but is far less considered in connection with scientific research teams – with the possible exception of psychological screening for those applying to be astronauts (Vakoch, 2011). This consideration has obvious consequences, as it applies constraints to the openness of such scientific work. Future teams are usually based on past teams, personal experience or personal recommendations about whether a researcher is a ‘good chap’. Not only does this practice usually exclude women but it also shapes the age, nationality and even political and religious profiles of those involved in scientific work. Twenty years after the Silver Hut expedition, John West was worried about bringing Dr Karl Maret on the American Medical Research Expedition to Everest (AMREE) because he dressed like a ‘hippie’, while women members would ‘create additional tensions’ (West, 1985: 23, 30). These inclusionary and exclusionary practices are by no means limited to field site work, but perhaps such spaces allow a more explicit discussion of what makes a ‘good chap’ than documents relating to hiring decisions in university or commercial laboratories, and therefore make these assumptions visible to historians in useful ways.

Silver Hut – laboratory, field station, field site and expedition – also complicates our ideas of isolation in scientific research. Some of the ‘work’ of a laboratory, it has been argued, is to delineate in- and out-groups, exoteric and esoteric categories of expertise and knowledge (Fleck, 1979[1935]; Kohler, 2008; Shapin, 1988). Of course, in a sense a Himalayan research laboratory could not be more exclusive. Expense, geography, physical ability, inclination, red tape, immigration and access to the right circles to get an invitation all act to create an extremely tight and esoteric group of elite practitioners able to gain access to this particular home for science. These limitations also pose a challenge to the demand that scientific work should be replicable to be trustworthy; that is a topic for another paper. It is not novel to point out that even the most remote laboratory is in fact connected to an intricate web, perhaps by roads, rail, seaplane, telegraph or Internet, and that this interconnectedness is often crucial to the truth value of work done in the laboratory (Becker, 2009; Gieryn, 2006). This is clearly visible for INPHEXAN in Antarctica, relying on existing military and expeditionary personnel and their buildings, collaborating in creating new spaces for science, sending samples back by boat and by plane and using sledges and seaplanes to travel around the isolated continent. Silver Hut may seem less connected, but it had a regular postal service, radio and telegraph, and Hillary flew in and out multiple times. Field stations can have very porous barriers, and sometimes these allow serendipitous results: some of the most interesting and yet unplanned research in the Silver Hut was conducted by Pugh on Man Badhur, a Nepalese pilgrim who was living locally and walked, barefoot in thin clothes and without tent or sleeping bag, to visit the Silver Hut, much to the amazement of the scientists in residence (Pugh, 1963).

## Never artificial? The afterlives of field sites

Historians have not adequately (or in many cases, even peremptorily) considered the afterlives of field laboratories or of other sites of scientific knowledge production; a notable exception here is Amanda Rees’ (2006) work, which provides a good example of the point that the presence and activity of research and observers inevitably changes field sites (see also Livingstone, 2003). Even when the research or other scientific activity of a site is terminated and a hut or a room turned to pedagogic, touristic or domestic use, the nature of the building and the remembrance of its work can still have powerful social and cultural influences. The Capanna Margherita’s identity changed somewhat over time – it went from a pioneering outpost, to a convenient site for weather observations, to a ‘staging post’ for higher expeditions. Silver Hut has undergone an even more dramatic transformation.

Obviously, the many ‘Silver Huts’ – the field site on the Mingbo Glacier and its environs, the mobile laboratory and other infrastructure, and the collection of data, experimental reports, publications and personal (embodied) experiences – have different fates. The first apparently vanishes with the removal of the human participants, the second is repurposed and the third becomes codified into facts. Both the Silver Hut and the Green Hut were dismantled and left in the Himalayan region for the use of future climbers and physiologists.^11^ Green Hut appears to have become used exclusively for domestic and shelter purposes, while Silver Hut remained a home for science, donated to the Indian Government. The availability of a high-altitude research laboratory was one of the stimuli for the founding of the Indian Defence Institute of Physiology and Allied Sciences (DIPAS), which made heavy use of Silver Hut through the 1970s. At the same time as it became a site for (largely military-funded) research, it was also reinvented as a site of secular pilgrimage for altitude researchers (Dass and Bhaumik, 2012). The Silver Hut became an important passage point – to use a phrase Latour applied to the new modern, 19th-century laboratory– for Indian researchers who wanted to work on topics relating to extreme and altitude physiology. Time at Silver Hut functioned both as a proof of status and as a way to connect to what is represented as a line of heritage directly back to the ‘seminal’ studies of Pugh’s team.

What is most extraordinary about this reification of the laboratory is that the Silver Hut itself has been moved (Dass and Bhaumik, 2012). The Silver Hut was reassembled at the Himalayan Mountaineering Institute, Darjeeling, at Chowri Kang, Sikkim, around 4500 m above sea level, barely 50 m higher than the Capanna Margherita (West, 2001). That the (slightly tattered) remains of a field laboratory can maintain a status despite total dislocation from its associated field *site* says something rather complicated about how experimental practice can alter the nature of physical objects, turning them into both instruments and relics. This particular prioritization of the man-made buildings of science over the specific field site itself is shared by the original participants of the Silver Hut expedition, who in 2000, to celebrate the 40th anniversary of the expedition, returned not to the Mingbo Glacier but to the Hut in Chowri Kang. As John West (2001) wrote, ‘Jim’s [Milledge] plan was for the “Silver Hut survivors” to trek in to the site, to review some of the results of the expedition, and to revive old memories’ (p. 311).

This afterlife belies the fact that the specific geographical location of the original Silver Hut was absolutely part of its status as an appropriate home for science. Without its high-altitude location, without its access to mountains and its localization somewhere that would suit Yeti hunts and climbing expeditions, it was not worth funding and could not claim a special status as a maker of knowledge about altitude. Researchers looking at such field sites frequently refer to a mountain or an Antarctic plain as ‘a (natural) laboratory’, in both private and public correspondence, in scientific publications and funding proposals. This is, in both rhetoric and practice, a very different matter from the field site that is intended to offer authentic scientific insights into a ‘natural’ world, where natural is supposed to oppose ‘artificial’, taken as a system affected by the interference of people (Rees, 2006). But it is also quite different from the ‘place-based research’ achieved through zoological research stations, which are, as discussed above, often represented as borderlands (De Bont, 2014). In the Silver Hut, the Mingbo Glacier actively intervened in the scientific work, providing not only physical space or collectable data but also environmental conditions and variables that were used to produce experimental facts.

Field stations, like Silver Hut, can become so important that they function as the core of a network in their own right. Partly this is achieved by controlling biological samples; one of the justifications for fieldwork is that it generates material objects (e.g. alveolar air samples, seal blood samples) that are unobtainable elsewhere. But because of the distribution networks, access to these precious materials is not limited to people who have physical access to the sites of scientific work but rather also to those with the right personal or institutional connections (or funds). Just as important, field sites also produce trained (disciplined) scientists, and it is through these workers, as much as through the export of publications or material objects, that the field station becomes interconnected with other research sites. In the case of Silver Hut, it was connected through personnel to the next ‘world’s highest laboratory’, erected twenty years later on Everest.

The AMREE in 1981 was led by ex-Silver Hut resident John West, along with two of the younger members of the team, Milledge and Lahiri (West, 1984). In an account of this expedition in the journal *Science*, West (1984) explicitly refers to ‘the natural laboratory of the mountain itself’ as a better option for studying altitude physiology than the barometric chamber (p. 784); while Silver Hut made do with Mingbo and Makalu, the AMREE secured permission to go to Pugh and Hillary’s original destination: Everest. This expedition also had prefabricated laboratory buildings, one at Everest Base Camp and the other on the Western Cwm. The Base Camp laboratory was slightly lower than the original Silver Hut, at about 5400 m above sea level, while the Western Cwm laboratory was significantly higher at 6300 m. AMREE’s plans also included an attempt on the summit of Everest and series of physiological self-measurements to be taken as high as possible. In the end, Dr Chris Pizzo took the highest biomedical measurement possible on earth, sampling his own alveolar air and recording his electrocardiogram (ECG) and ventilation rate on the summit, at 8848 m (West, 1998: 333).

The structure of the expedition was clearly influenced by Silver Hut, but so too was the physical infrastructure, with the Base Camp Laboratory, based on the original Silver Hut design, breaking down into pieces (thirty ‘flanges’) that could be carried as a single load. The laboratory, which weighed 700 pounds and was 7 ft wide, 15 ft long and 7 ft high, could be assembled in less than two hours. Initially, the plan had been for this laboratory to be installed at the higher Western Cwm site, but a severe storm discouraged the team, and instead they created a lighter-weight laboratory at Camp 2 using a plywood floor and aluminium frame covered with fibreglass blankets, making it look more like a tent than the almost shipping container-like Base Camp Laboratory (West, 1984: 330–331). These laboratories, too, were recycled; the team sold their domestic spaces and other equipment to a Canadian team attempting Everest in 1982, while the dismantled Base Camp Laboratory was stored in Periche, Nepal, and West asked the American Physiological Society to let its members know they should contact him if they wanted to use it (West, 1985: 56).^12^

## Universalizable and unique homes for science

Most homes for science have to negotiate one particular binary conflict: the tension between uniqueness and universality. That is, the workers in each laboratory, field station and so on must make a case for the particular value of that site as a space in which reliable knowledge can be produced; at the same time, such knowledge needs to be universalizable.^13^ Mosso’s work on fatigue was of relevance specifically to mountaineers, but it also informed military and industrial practice; Pugh’s studies of Antarctic cold reactions and Pizzo’s blood and air samples helped explorers, but they also fed into work on the survival of premature babies and the needs of future space travellers. Historians face a similar challenge; while the most anthropologically or ethnographically informed studies of the homes of science may just provide detailed accounts of unique places, most of us attempt to universalize – or at least generalize – from our case studies. We are expected to demonstrate, like Aubin, the three ways in which a mountain can be made into a laboratory or, like Kohler, insert a third category of ‘borderland’ between the existing assumptions of ‘field’ and ‘laboratory’. The danger of such generalizations is that they become scripts; they dictate the terms of the future study – for example, the distinction between ‘laboratory’ and ‘field’ is difficult to avoid, even where, as in this article, part of the argument is that such a distinction can be unhelpful.

The reason these challenges matter is because the homes for contemporary science are becoming extraordinarily complex – indeed, they always have been. To return to Antarctica, we find a site that blurs almost all categorical assumptions: field, site, laboratory, instrument, nature and artifice are all blended into a single home for the production of scientific knowledge. The IceCube Neutrino Observatory is based nominally at the Amundsen-Scott Pole Station – and already that location layers on some complexity; founded by the American government as part of the International Geophysical Year in 1956 (and host to some of the INPHEXAN work), this sometime home for science is named after a British and a Norwegian explorer, tapping into the nostalgia of the ‘Heroic Age’ of exploration. The base also is not a static object; it has been remodelled and rebuilt several times so that although it has been continuously inhabited, it is not quite a relic in the same form as Silver Hut. Like Silver Hut it was built on a glacier, and like Silver Hut it has been mobile – as the glacier moves so does the base itself. As with all the spaces considered in this article, it is the result of complex collaboration and co-funding: nominally a university laboratory, it is a very long way from its parent institution, the University of Wisconsin, and boasts a huge list of international co-collaborators and funders (IceCube South Pole Neutrino Observatory, 2014). But, of course, it also directly relies on the Amundsen-Scott Station, and in turn on the landing strips, ports of call, support stations and established supply chains that make survival in the Antarctic possible.

To add to this multiplicity, the Observatory itself is not a simple piece of physical infrastructure but a combination of instrument and landscape, a cyborg object, perhaps. The instrument consists of an adapted cubic kilometre of the polar ice itself. On top of this vast structure sits a comparatively small ‘laboratory’ space; the Observatory is made functional by the drilling of deep holes in the ice, into which are threaded extremely sensitive light sensors. Tiny flashes of light caused by neutrinos hitting the ice pack and interacting with frozen water molecules are detected by the strings, so in this design the ice itself is clearly being instrumentalized. Here, there is an almost seamless shift across the artifice/nature, or man-made/natural boundaries, from an (imagined) natural landscape and its intrinsic properties and facts to an instrument embedded in a laboratory-scape.

This article is not an attempt to come up with yet another definition or characterization of ‘the field site’, instead it is arguing, simply, that while ‘field site’ is a useful *pragmatic* descriptive designation, and an actors’ category, it is not a useful *analytic* category. For example, the mobility and reification of the Silver Hut field laboratory are not suggested as a special feature of work in field sites; there are field laboratories that cannot meaningfully be moved from their field site (for example, the IceCube), while there are non-field laboratories that can be successfully moved across campuses or continents, and historic laboratories (such as the Cavendish in Cambridge) that, although no longer functioning in their original context, are sites for ‘scientific pilgrimage’. In some instances, specific field sites may prove particularly useful to the historian because they make practices clearly visible – and that is certainly the case for the neglected homes for science considered here. For example, we have been discovering, since Shapin’s (1989) seminal work on ‘invisible’ or ‘behind-the-scenes’ activity, that the role of auxiliary and support staff is crucial to the production of scientific facts. This is as true of natural philosophical laboratories or Royal Society demonstration theatres as it is of huts on glaciers; yet the latter might make this truth more visible. The homes for science discussed here particularly reveal the role of, for example, indigenous peoples or the media (most European mountaineering expeditions of the 20th century included some sort of arrangement whereby one media outlet got privileged access to news releases and updates).

Field sites therefore expose the broader supporting infrastructures of scientific work. Although these are sometimes stripped from traditional scientific publications, they remain accessible to historians because of the way field sites interact with other social spheres – most notably exploration, sport and the military. Work in extreme environments taps into notions of endurance and/or the glamour of exploration, which in turn genders them (Oreskes, 1996). This allows more descriptive and less objective accounts of the work done to survive even in relatively formal publications or presentations and even more so in editorials, retrospectives, interviews and autobiographical pieces (Hevly, 1996). The visible participation of non-scientists draws our attention to other sources – such as articles about exploration or advertisements in climbing magazines – where Sherpas, explorers, funders and non-human objects like the weather or sled-dogs can all be seen explicitly performing scientific work. It is also the case that the physical infrastructures of these laboratory spaces are more frequently discussed than is common in studies conducted in more ‘traditional’ research spaces; usually entirely tacit in most experimental write-ups, it is possible to find building instructions for walls, doors, heating systems and so on, within or alongside the traditional published accounts of data generation and interpretation (Halzen and Klein, 2010). If we want to understand the homes of science, we need to look to where the ‘home’ is fully described and discussed.

It is clear that for physiological research in extreme spaces, military and sporting infrastructure is important. While the role of military money and pressures in 20th century science is hardly a new discovery, the role of sport is almost entirely invisible (with some exceptions, mostly relating to drugs and altitude; for example, Heggie, 2008; Tracy, 2012; Wrynn, 2004, 2006), and while explorers are crucial actors in earlier scientific networks, they barely feature in existing histories of 20th-century work (Heggie, 2014). It may be that the heavy involvement of sport and exploration are unique features of field sites, but we are not yet in a position to make that assertion, and it seems unlikely since the few works on recent experimental physiology, such as those by Andi Johnson (2013) or Robin Scheffler (2011, 2015), suggest that sport’s relation to all the homes of science is an area ripe for reconsideration.

There are differences here for the historian to document; there is clearly a difference between a doctor who is both experimenter and test-subject taking a sample of expired alveolar air, with great difficulty, in extreme weather conditions, at high altitude in the Himalayas, and a sample taken from a volunteer student in a sea level, environmentally controlled university physiology laboratory. What is not clear is that the best way to categorize this difference is by declaring one practice to be field science and the other not-field science. The ease with which Aubin’s characterisation of a mountain site can be repurposed for all sorts of homes of science, in all sorts of spaces, as well as the ontological complexities and genre-blurring characteristics of the sites considered in this article, all suggest that we need a richer language of description to understand the practice of modern science. If, then, we cannot appeal to our existing models to explain the difference between an Everest and a Berkeley air sample – that is, to our understandings of discipline, metrication and the assumed dominance of experimental non-fieldwork – to what can we appeal?

Perhaps the difference is economic. In the case of the air samples, one was vastly more expensive to collect than the other, and because of the challenges of the space of collection, one is much rarer than the other. Does this cost and rarity require different systems and rhetorics of justification? Does one sample have a stronger ‘truth value’ because of its rarity (or vice versa)? Perhaps the most significant variation is that between self-experiment and other experiment, which creates different identities for the scientists and samples involved, but which is effectively independent of the field or non-field status of the experiment? Or, as Kuklick (2011) has hinted, could the value of the sample be affected by ideas borrowed from sport, exploration, and colonialism, about heroism, individual sacrifice and intellectual (White, male) dominance over nature (Powell, 2007)?^14^ Whatever the answer, we will not find it without reconsidering all the homes of science.

## Notes

1. The gendered nature of this work is explicit; the few female visitors to the Faulhorn, at least for most of the 19th century, seemed to be limited to enterprising hikers and hotel staff rather than active researchers.

2. I have only read this work in its 1898 English translation. The English edition contains barometric chamber research that is not present in the earlier Italian version (Di Giulio and West, 2013).

3. Although Edmund Hillary claimed that the hut was based on his own design, the archives of the Silver Hut expedition show this is unlikely to be the case (Tuckey, 2013: 257).

4. The expedition is sometimes also referred to as the Anglo-American Physiological Expedition to Antarctica. LGCE Pugh Papers MSS 0491. University of California at San Diego: Mandeville Special Collections Library (hence, LGCE Pugh Papers), Box 39, Folder 13 – Contract Nonr-222(5) between the ONR and the Regents of the University of California, Summary Report of Operations in Antarctica (INPHEXAN: International Physiological Expedition to Antarctica) 16 June 1958.

5. LGCE Pugh Papers MSS 0491. Contract Nonr-222(5), cited in note 4.

6. LGCE Pugh Papers. Box 39, Folder 12 – Letter Pugh to Chief of Naval Research, Washington, 31 July 1957.

7. LGCE Pugh Papers. Box 1, Folder 2 – Report of Work During the Period April 1958–1959.

8. LGCE Pugh Papers. Box 8, Folder 42 – Letter to Miss J Walker of the Commonwealth Relations Office 21 November 1957; Folder 48 – Letter to Douglas Side of the Mt Everest Foundation 21 November 1957.

9. It also says something about the pivotal role of Sherpas in the expedition; whether this is to be read as a celebratory statement about their contributions or as a way of slighting their perceived ‘childishness’ or a scientific attitude (or both), is unclear.

10. John West Papers, MSS 0444, University of California at San Diego: Mandeville Special Collections Library (hence, West Papers), Box 8, Folder 4 – Letter West to ‘Hackett’, 5 December 1988.

11. This ‘recycling’ of laboratory equipment, up to and including the laboratory itself, is a common feature of expeditionary science and is examined in more detail elsewhere. (Heggie, 2012).

12. West Papers, Box 1, Folder 15 – Letter West to OE Reynolds, 28 December 1981.

13. One exception to this role may be some sites related to ecological and environmental work, where it is possible to represent the knowledge produced as valuable because it is entirely context-specific. Even in these situations, however, parts of the work – such as the methodology, if not the conclusions – are expected to be applicable to other sites, and other questions.

14. For example, the very willingness to climb to high altitude was read as evidence of Western modernism, in opposition to Central Asian superstition, by the first European explorers in the Himalayas (Hansen, 2001).
